# Glacial Periods

**Published:** 1881-06

**Authors:** 


### GLACIAL PERIODS.

Mr. H. B. Norton, in a recent lecture before the Kansas Academy of Science, gave some interesting calculations respecting the glacial epochs through which the North American Continent has passed, based laigely upon the theories of Croll and Geikie. In his closing paragraph he remarks : "It thus seems probable that there have been many glacial periods in each hemisphere, and that the ocean, like a mighty pendulum, vibrates from pole to pole through vast but regular periods. It is not necessary to suppose a cataclysm at the end of each period, as some of the earlier writers did; but rather an insensible drainage of waters, which so gradually submerges the land and pushes the human race before it as hardly to be perceptible in the course of generations, even uncovering new continents, and opening up fresh fields and pastures new to human industry when the old are exhausted. The Southern hemisphere is now undergoing the slow refrigeration of its long winter. This began about 6,500 years ago ; it will end about the year 4870. It has passed its middle, but not its culmination, even as the greatest average cold of our ordinary winter is nearer the vernal equinox than the winter solstice. It is probable that 2,000 years from now the southern continents will be still more deeply deluged ; the Antarctic ice-cap glaciers will have extended several hundred miles to the northward, and the glaciers which have already appeared among the Andes will have covered the plateaus of Patagonia and Chile. Nevertheless we need not expect that mankind will then witness the utmost possible degree of refrigeration, because the ellipticity of the earth's orbit is now less than it has been at certain periods in the past, and will be again in the remote future."—*Ibid.*

